# Supplementary material for: Motor neurons control blood vessel patterning in the developing spinal cord
Source: Nat Commun. 2017 Mar 6;8:14583. doi: 10.1038/ncomms14583 (PMC5343469; doi:10.1038/ncomms14583)
Supplement: Supplementary Information — Supplementary Figures, Supplementary Tables. [file ncomms14583-s1.pdf]

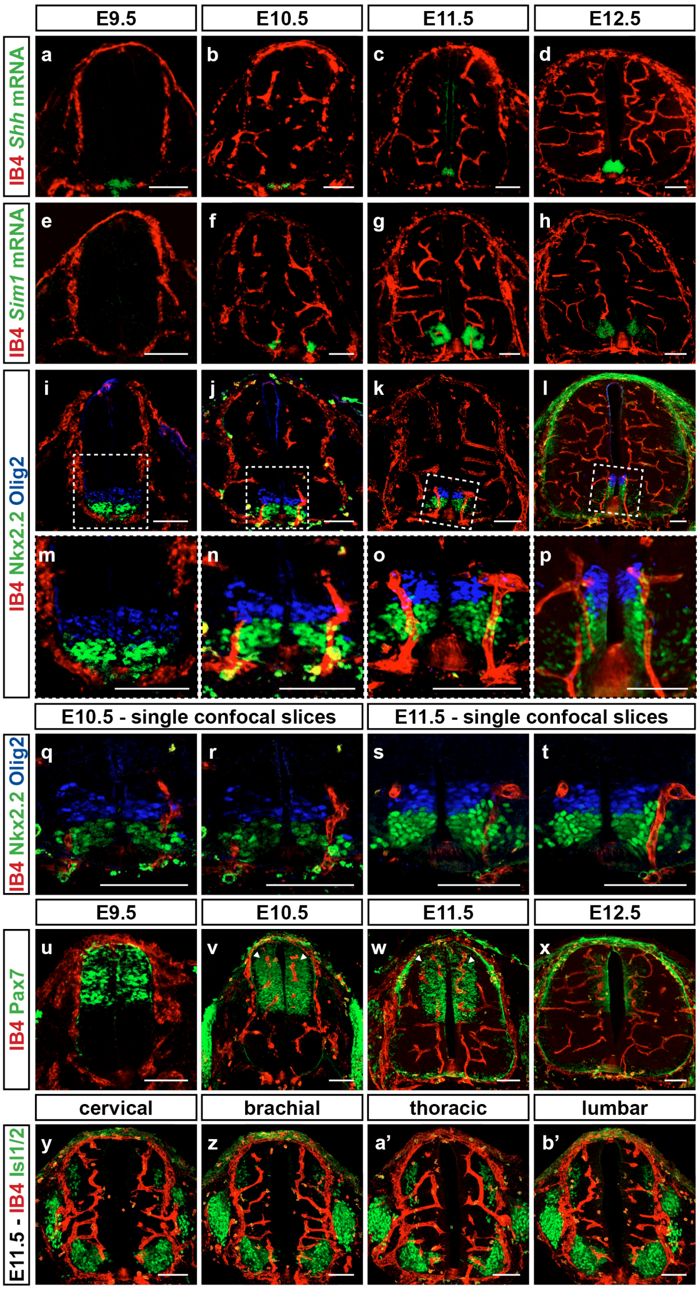

Supplementary Figure 1: Blood vessel patterning in the developing spinal cord is highly stereotypical. (**a-h**) Representative images of mouse embryo SCs at the developmental stages indicated showing blood vessels (IB4$^+$) combined with ISH for *Shh* (**a-d**) to label the floor plate, or with *Sim1* (**e-h**) to label post-mitotic V3 interneurons, revealing that blood vessels avoid the floor plate and sprout through *Sim1*$^+$ V3 interneurons. (**i-l**) Representative immunostainings of SCs at the developmental stages indicated showing blood vessels (IB4$^+$), p3 neuronal progenitor domain (Nkx2.2$^+$) and pMN neuronal progenitor domain (Olig2$^+$). (**m-p**) Higher magnifications of insets in (**i-l**). (**q,r**) Representative single confocal slices of the image shown in (**n**). (**s,t**) Representative single confocal slices of the image

shown in (**o**). Note the close contact of ventral blood vessel sprouts with p3 and pMN neuronal progenitor domains. (**u-x**) Representative images of blood vessels ($IB4^+$) combined with immunostaining for Pax7 to label dorsal neural precursors from E9.5 till E12.5, showing that blood vessels branch dorsally in the $Pax7^+$ neuronal domain (white arrowheads). (**y-b'**) Representative images of E11.5 SC sections sampled at cervical, brachial, thoracic, and lumbar levels showing endothelial cells ($IB4^+$) and post-mitotic MNs ($Isl1/2^+$). Note that a similar vascularisation pattern around MN columns occurs at all SC levels. Scale bars 100 μm.

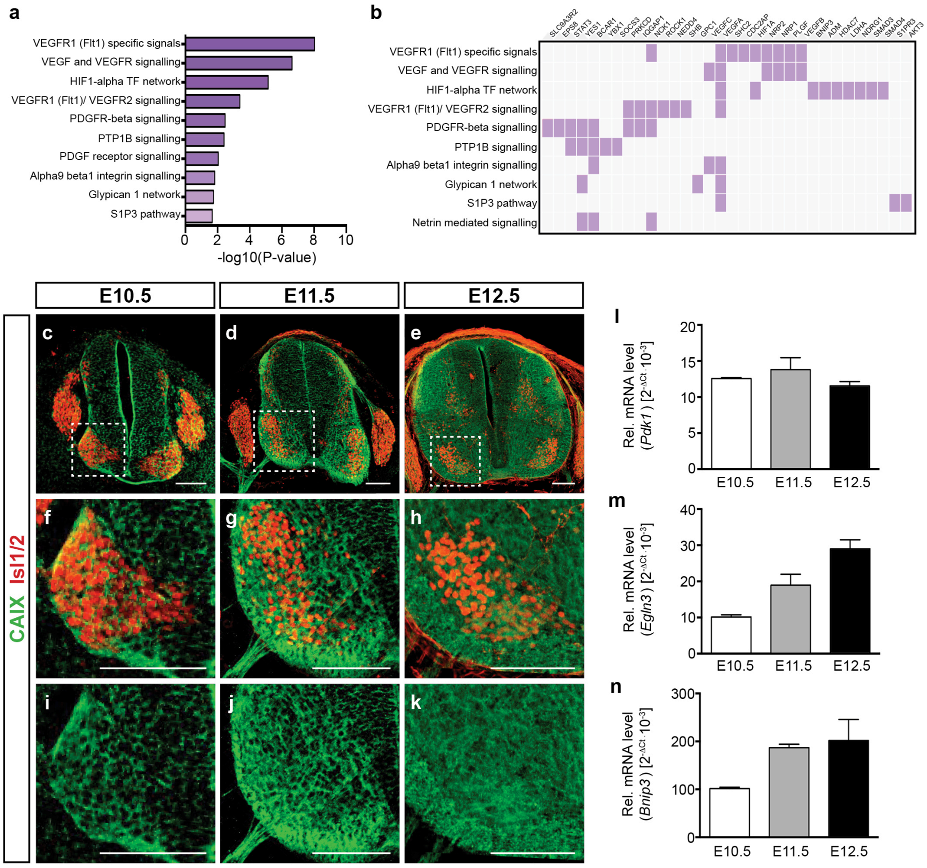

Supplementary Figure 2: HIF1α regulates VEGF expression in motor neuron columns. (**a**) Enrichment analysis for signalling pathways associated with potential VEGF regulators that are overexpressed or expressed in E11.5 MNs using *Enrichr*. (**b**) Clustergram of the potential VEGF regulators grouped by signalling pathways. (**c-e**) Representative immunostainings of embryonic SCs at the developmental stages indicated, showing post-mitotic MNs (Isl1/2^{+}) combined with immunostaining for CAIX (a gene regulated by Hif1α). (**f-k**) Higher magnifications of insets in (**c-e**) showing either the merge immunostaining (**f-h**) or just CAIX immunostaining (**i-k**). Scale bars 100 µm. (**l-n**) qRT-PCR analysis of prototypical HIF target genes (*Pdk1* (**l**), *Egln3* (**m**) and *Bnip3* (**n**)) in microdissected MN columns at E10.5, E11.5 and E12.5. Data is represented as mean±s.e.m.. n = 2 individual experiments done in triplicates.

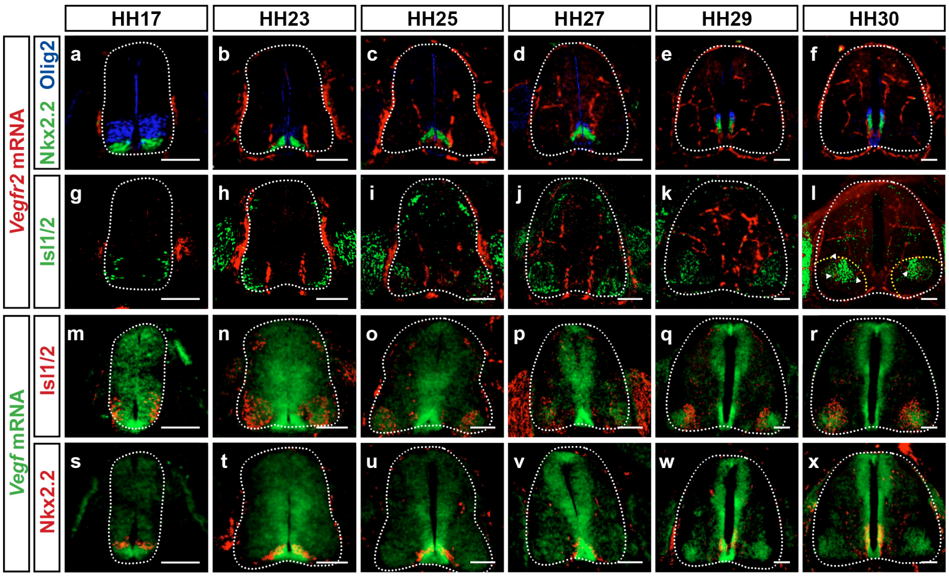

Supplementary Figure 3: VEGF expression and spinal cord vascularisation in chicken embryos is analogous to mouse embryos. (**a-f**) Representative images of SCs from chicken embryos from HH17 till HH30 showing blood vessels (labelled by ISH for *Vegfr2*), p3 neuronal progenitor domain (Nkx2.2^{+}), and pMN neuronal progenitor domain (Olig2^{+}). **(g-l)** Representative images of the correlation of SC vascularisation pattern (blood vessels labelled by ISH for *Vegfr2*) with post-mitotic MNs (Isl1/2^{+}) at the same developmental stages as above. Note blood vessels stay outside the Isl1/2^{+} domain till HH30 (yellow dotted lines: MN columns; white arrowheads marking blood vessels in MN columns at HH30). (**m-r**) Representative images of ISH for *Vegf* combined with immunostaining for post-mitotic MNs (Isl1/2^{+}). (**s-x**) Representative images of ISH for *Vegf* combined with immunostaining for p3 neuronal progenitor domain (Nkx2.2^{+}) to show that *Vegf* is expressed in this progenitor domain (Nkx2.2^{+}) and in the floor plate (domain Nkx2.2^{-}, below Nkx2.2^{+}). Scale bars 100 μm.

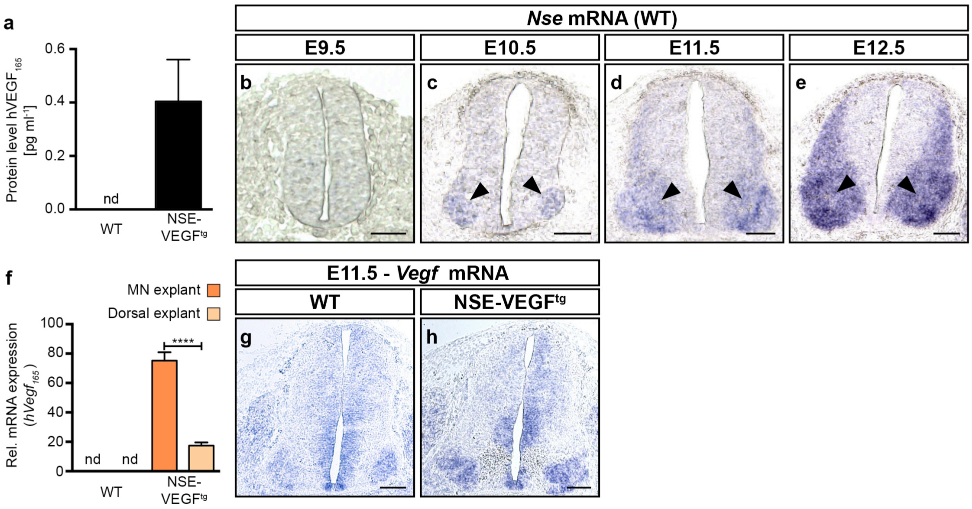

Supplementary Figure 4: NSE-VEGF^{tg} mouse embryos express high levels of hVEGF$_{165}$ in the ventral part of the spinal cord. (**a**) ELISA for hVEGF protein in SC lysates from E11.5 wildtype (WT) and NSE-VEGF^{tg} embryos. n = 3 individual experiments done in triplicates. nd = not detectable. (**b-e**) ISH for *Nse* in the developing SC from E9.5 till E12.5. Note the high expression of *Nse* in the ventral half of the SC (especially in MN columns (black arrowheads)). (**f**) qRT-PCR analysis of *hVegf$_{165}$* mRNA in microdissected MN columns and in areas of the dorsal SC of E11.5 WT and NSE-VEGF^{tg} embryos. n = 2 WT embryos and n = 8 NSE-VEGF^{tg} embryos. nd = not detectable. ****P<0.0001. (**g,h**) ISH for *Vegf* (*mVegf* + *hVegf*) revealed higher expression levels of total *Vegf* in NSE-VEGF^{tg} embryos (**h**) compared to WT (**g**). As at E11.5 NSE is mainly expressed in MNs, the slight increased expression of *Vegf* is probably *mVegf* and might be a secondary effect due to the NSE-driven h*Vegf* expression.

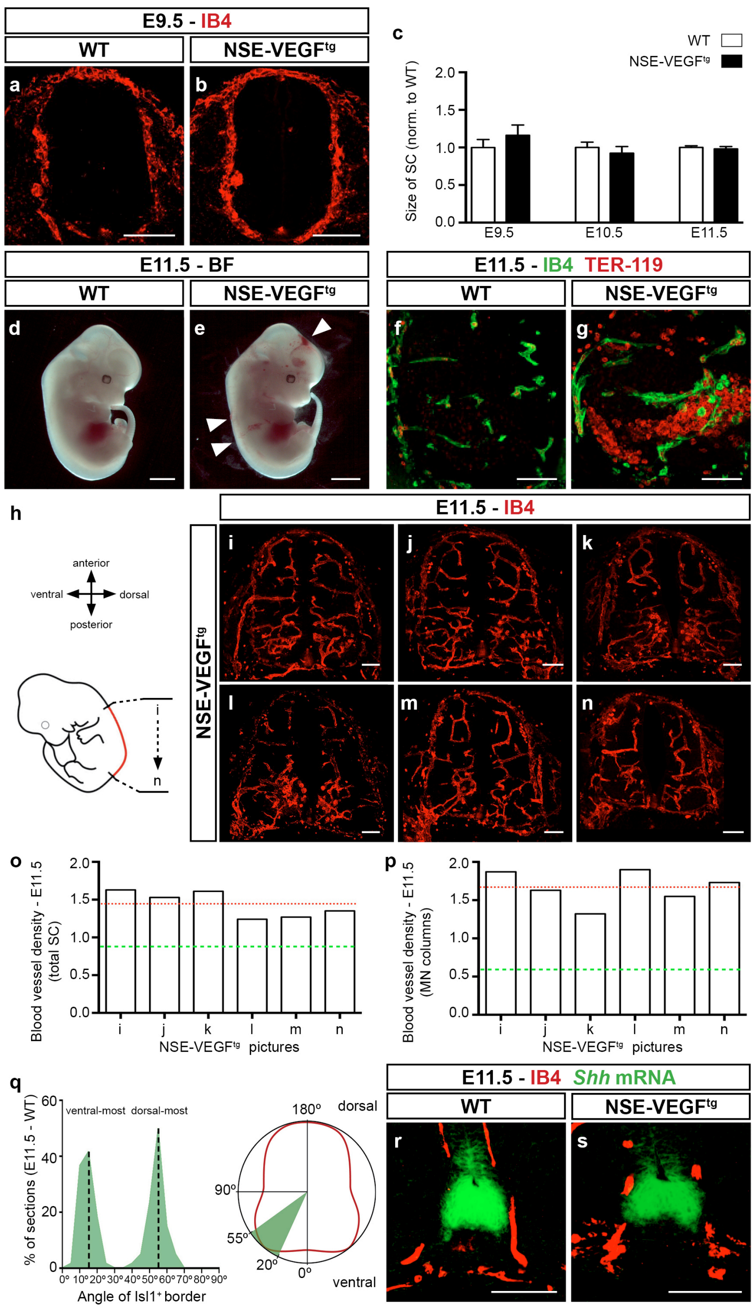

Supplementary Figure 5: NSE-VEGF^{tg} mouse embryos reveal comparable vascularisation defects throughout SC levels analysed. (**a**,**b**) Transverse SC sections stained for endothelial cells (IB4^{+}) at E9.5 in WT (**a**) or NSE-VEGF^{tg} embryos (**b**). (**c**) Analysis of SC size for WT and NSE-VEGF^{tg} embryos at E9.5 (n = 7 WT; n = 14 NSE-VEGF^{tg}; 3 different litters), E10.5 (n = 5 WT; n = 11 NSE-VEGF^{tg}; 3 different litters) and E11.5 (n = 15 WT; n = 41 NSE-VEGF^{tg}; 8 different litters). Data is normalised to the respective WT. P=ns. (**d**,**e**) Images of WT and NSE-VEGF^{tg} littermate embryos. White arrowheads point to signs of haemorrhages. Scale bars 1 mm. BF: bright field. (**f**,**g**) Double staining for blood vessels (IB4^{+}) and for the erythrocyte marker (TER-119^{+}) showing vascular leakage in the ventral half of the SC

in NSE-VEGF^{tg} embryos (**g**). (**h**) Mouse embryo scheme showing the region used in this study for analysing SC vascularisation. Sampling heights of SC sections shown in (**i-n**) are indicated. (**i-n**) Transverse SC sections at brachial and thoracic level, shown from anterior (**i**) to posterior (**n**) of the E11.5 NSE-VEGF^{tg} embryo shown in Fig. 3 (endothelial cells (IB4^{+})). (**o,p**) Quantification of blood vessel density in the entire SC (**o**) or within MN columns (**p**) for each panel shown in (**i-n**). Green dotted lines: average of WT embryos analysed; red dotted lines: average of NSE-VEGF^{tg} embryos analysed. (**q**) *Left*: Graph representing the measured angles, where the *ventral-most* border and the *dorsal-most* border of MN columns (Isl1^{+}) are located, taking as a 0º reference the floor plate. n = 6 WT embryos. *Right*: Scheme representing the angles where the core of MN columns (from 20º to 55º) is located with respect to the floor plate. Floor plate = 0º, Roof plate = 180º. (**r,s**) Representative images of blood vessels (IB4^{+}) combined with ISH for *Shh* showing that blood vessels do not invade the floor plate in WT (**r**) nor in NSE-VEGF^{tg} (**s**) embryos. Scale bars 100 µm.

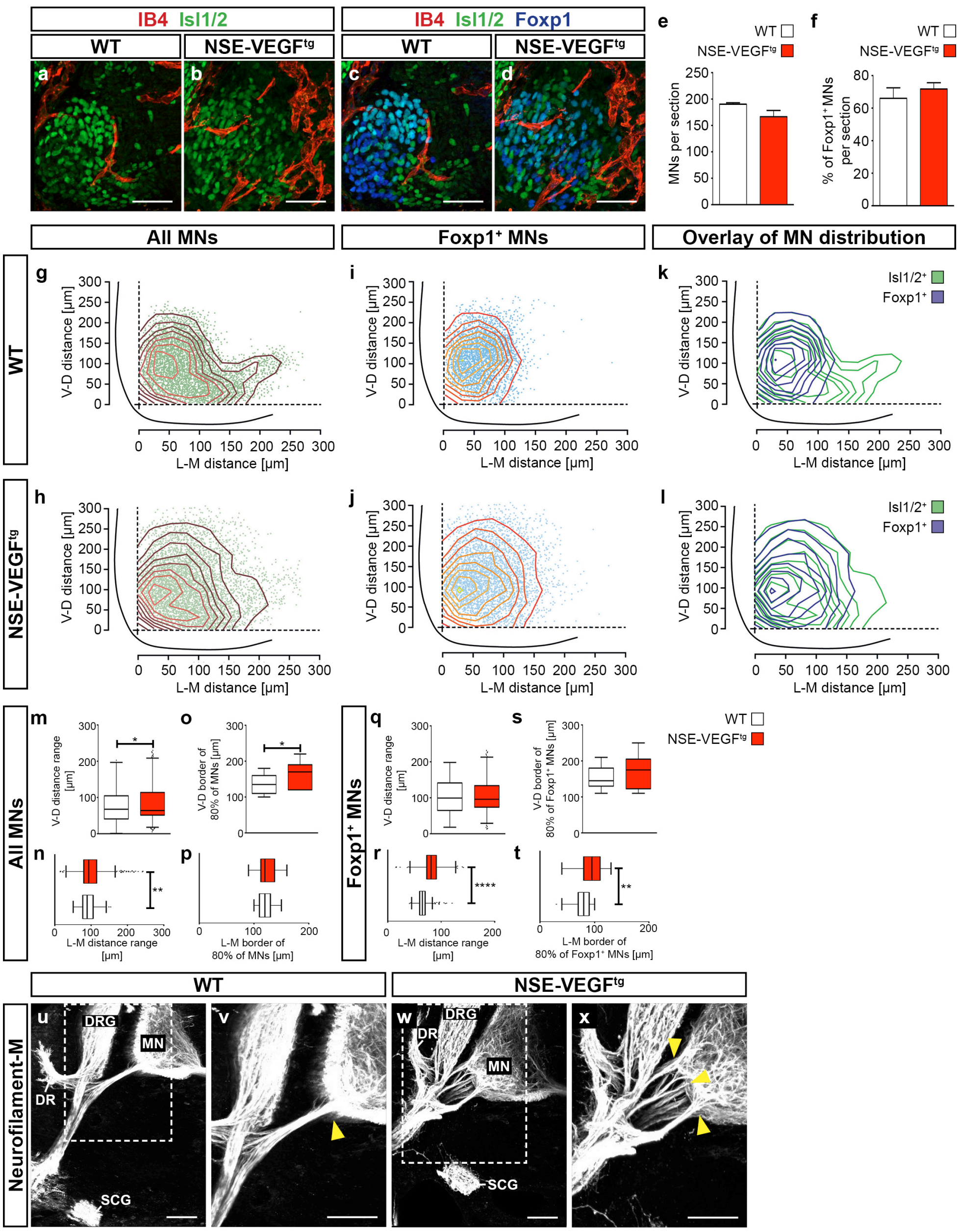

Supplementary Figure 6: NSE-VEGF^{tg} embryos show defects in motor neuron distribution. (**a,b**) Representative images of MNs stained for endothelial cells (IB4^{+}) and post-mitotic MNs (Isl1/2^{+}) of wildtype (WT) (**a**) and NSE-VEGF^{tg} embryos (**b**). (**c,d**) Triple immunostaining showing all MNs (Isl1/2^{+}), lateral MNs (Foxp1^{+}) and endothelial cells (IB4^{+}) in WT (**c**) and NSE-VEGF^{tg} embryos (**d**). (**e,f**) Quantification of total MN number (Isl1/2^{+}) (**e**), and percentage of Foxp1^{+} MNs (in respect to all MNs) (**f**) per section reveal no difference between NSE-VEGF^{tg} embryos and their WT littermates. n = 3 WT and n = 8 NSE-VEGF^{tg} embryos from 2 different litters. P=ns. Data is represented as mean±s.e.m.. (**g-j**) Density plots of lateral-medial (L-M) and ventral-dorsal (V-D) position of all MNs (Isl1/2^{+}) (**g,h**) or of Foxp1^{+} MNs (**i,j**) of WT (**g,i**) and NSE-VEGF^{tg} embryos (**h,j**). The lighter the contour colour the higher the

MN density. (**k,l**) Density plot overlays of all MNs and Foxp1$^+$ MNs for WT (**k**) and NSE-VEGF^{tg} (**l**) embryos revealing an abnormal and intermixed MN positioning in NSE-VEGF^{tg} embryos. (**m,n,q,r**) Boxplots of the distance range covered by the position of all MNs (Isl1/2$^+$) **(m,n)** or Foxp1$^+$ MNs **(q,r)** at the V-D (**m,q**) and L-M (**n,r**) axis of WT and NSE-VEGF^{tg} embryos. *P=0.0438 (**m**), **P=0.0032 (**n**), ****P<0.0001 (**r**). Mann-Whitney test was performed for (**m,n,q,r**). (**o,p,s,t**) Boxplots representing the border distance up to where 80% of cell density concentrates at the V-D (**o,s**) and L-M (**p,t**) axis for all MNs (Isl1/2$^+$) (**o,p**) or for Foxp1$^+$ MNs (**s,t**) of WT and NSE-VEGF^{tg} embryos. *P=0.0117 (**o**), **P=0.0046 (**t**). For (**g-t**) n = 3 WT embryos and n = 3 NSE-VEGF^{tg} embryos from 2 different litters were analysed. Analysis was performed at brachial level in E11.5 embryos from at least 6 sections per embryo. (**u-x**) Representative images of 500 µm thick vibratome sections stained for Neurofilament-M showing MN axons (yellow arrowheads) leaving the SC in WT (**u,v**) and NSE-VEGF^{tg} embryos (**w,x**). (**v,x**) show higher magnifications of insets in (**u,w**), respectively. DR: dorsal ramus; DRG: dorsal root ganglia; MN: motor neuron; SCG: sympathetic chain ganglia. Scale bars 100 µm.

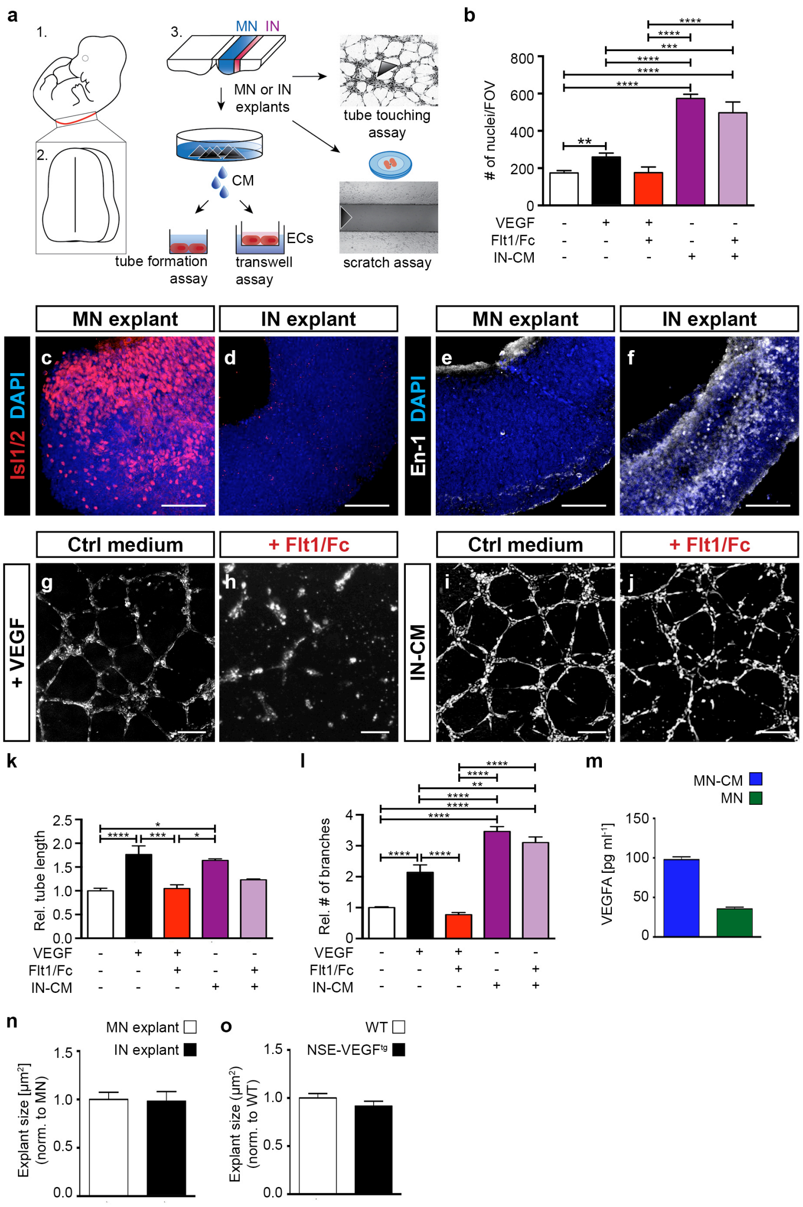

Supplementary Figure 7: *In vitro* neuro-angiogenic assays with MN and ingression site explants. (**a**) Schematic representation of the *in vitro* assays performed. Mouse embryos were dissected at E11.5 (1) and the SC isolated (2). Subsequently, an open book preparation was used to microdissect MN or ingression site (IN) explants (3). MN-conditioned medium (MN-CM) and IN-conditioned medium (IN-CM) was used for tube formation and transwell assays. In addition, MN and IN explants were used for tube touching assay or scratch assay. ECs: endothelial cells. (**b**) Quantification of transwell assay performed on HBMECs facing control (ctrl) condition, 50 ng ml^{-1} VEGF with or without 1 µg ml^{-1} Flt1/Fc, or IN-CM with or without 1 µg ml^{-1} Flt1/Fc. FOV: field of view. n = 3 individual experiments done in triplicates. **P=0.0027, ***P=0.0005, ****P<0.0001. (**c,e**) MN explants express the marker for post-mitotic MNs (Isl1/2^{+}) (**c**) but not the neuronal marker for V1 domain (En-1) (**e**).

(**d,f**) IN explants do not express Isl1/2$^+$ (**d**) but as expected express En-1$^+$ (**f**). (**g-j**) Representative images of tubular-structures formed by HBMECs in conditions of 50 ng ml^{-1} VEGF-treatment with (**h**) or without (**g**) 1 µg ml^{-1} Flt1/Fc, or IN-CM with (**j**) or without (**i**) 1 µg ml^{-1} Flt1/Fc. Scale bars 100 µm. (**k**) Quantification of tube-length. *P=0.0102 (ctrl vs. IN-CM), *P=0.0216 (VEGF+Flt1/Fc vs. IN-CM), ***P=0.0002, ****P<0.0001. (**l**) Quantification of number of branches. **P=0.0033, ****P<0.0001. For (**k**) and (**l**) n = 4 individual experiments done in triplicates. (**m**) ELISA for VEGF protein in MN-CM and MN lysate from E11.5 mouse SC. n = 3 individual experiments done in triplicates. (**n,o**) Quantification of the explant size (area) of MN and IN explants (**n**) as well as for MN explants from NSE-VEGF^{tg} mouse embryos and their wildtype (WT) littermates (**o**). n = 45 MN explants and n = 26 IN explants from 3 independent experiments. n = 12 MN explants per condition (WT and NSE-VEGF^{tg}) from at least 3 independent experiments. P=ns. One-way ANOVA using Tukey's multiple comparisons test was performed for (**b,k,l**) and P Values were adjusted to account for multiple comparisons. Data is represented as mean±s.e.m..

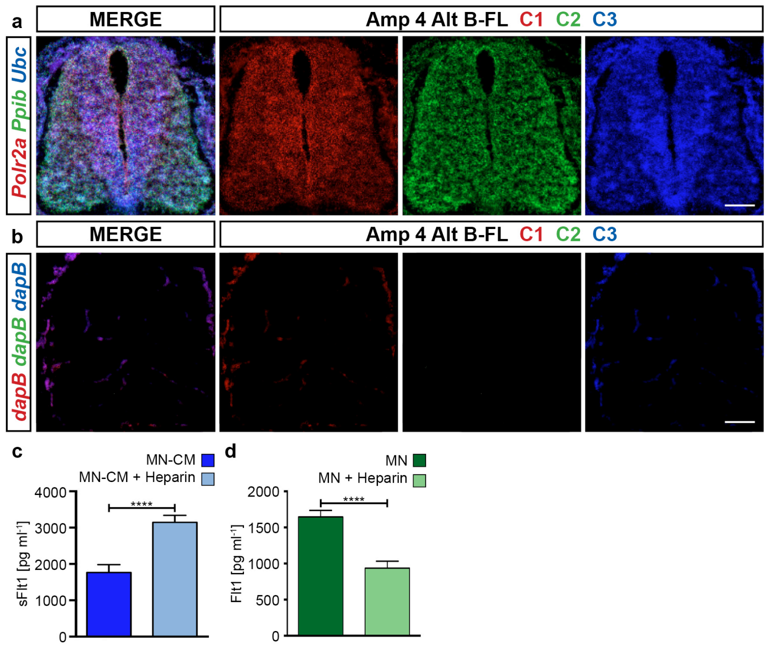

Supplementary Figure 8: sFlt1 is expressed by motor neurons and associated to the ECM of motor neuron columns. (**a**,**b**) Validation of RNAscope® Multiplex Fluorescent Assay. Fixed frozen transverse tissue sections of E11.5 mouse SC were hybridised with probes for *Polr2a, Ppib and Ubc* in multiplex fluorescent format as positive controls (**a**). Fixed frozen transverse tissue sections of E11.5 mouse SC were hybridised with a probe in multiplex fluorescent format for the bacterial gene *dapB* as negative control (**b**). Scale bars 100 µm. (**c**) ELISA for sFlt1 protein in MN-conditioned medium (MN-CM) of E11.5 mouse SCs with or without 10 U ml^{-1} heparin treatment for 20 h. n = 3 individual experiments done in triplicates. ****P<0.0001. (**d**) ELISA for Flt1 (mFlt1 + sFlt1) protein in MN explant lysates from E11.5 mouse SCs with or without 10 U ml^{-1} heparin treatment for 20 h. n = 3 individual experiments done in triplicates. ****P<0.0001. Data is represented as mean±s.e.m..

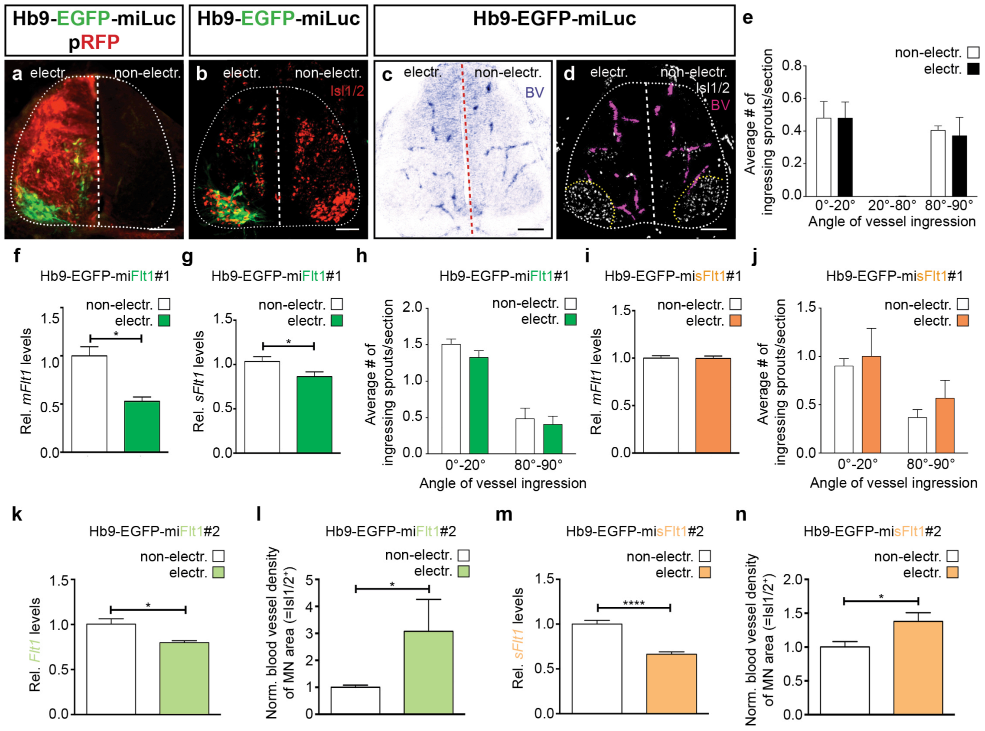

Supplementary Figure 9: Hb9-miRNA constructs specifically target motor neurons. (**a**) Representative image of *in ovo* co-electroporation of the control targeting vector (Hb9-EGFP-miLuc) with a pRFP-construct under the control of β-Actin promoter confirming specific targeting of MNs (green) as well as unilateral targeting of the chicken SC (red). (**b**) Representative image of Isl1/2 immunostaining in electroporated SC (Hb9-EGFP-miLuc) showing that targeted cells (green) are Isl1/2^{+} MNs. (**c,d**) Representative images of Hb9-EGFP-miLuc electroporated chicken embryos showing blood vessels (BV) labelled by ISH for *Vegfr2* (**c**) or traced in purple in (**d**). Scale bars 100 μm. (**e**) Quantification of the angle of ingression of blood vessel sprouts showing no differences between the Hb9-EGFP-miLuc electroporated side and the non-electroporated one. n = 8, P=ns. (**f,g**) qRT-PCR analysis confirms downregulation of expression levels of m*Flt1* (*P=0.0105) (**f**) as well as of *sFlt1* (*P=0.0405) (**g**) in MN columns from the Hb9-EGFP-miFlt1#1 electroporated side compared to the non-electroporated. For (**f,g**) n = 3. (**h**) Quantification of the angle of ingression of blood vessel sprouts in the ventral half of the SC before (0º-20º) or after (80º-90º) MN columns. n = 11, P=ns. (**i**) qRT-PCR analysis reveals no changes in expression levels of the membrane-bound form of *Flt1* in MN columns from the Hb9-EGFP-misFlt1#1 electroporated side compared to the non-electroporated. n = 4, P=ns. (**j**) Quantification of the angle of ingression of blood vessel sprouts in the ventral half of the SC before (0º-20º) or after (80º-90º) MN columns. n = 12, P=ns. (**k**) qRT-PCR analysis showing downregulation of *Flt1*

levels in MN columns from the electroporated side compared to the non-electroporated one in Hb9-EGFP-miFlt1#2 embryos. n = 3, *P=0.0369. (**l**) Quantification of blood vessel density in MN columns shown as ratio between electroporated and non-electroporated side in Hb9-EGFP-miFlt1#2, n = 8, *P=0.0266. (**m**) qRT-PCR analysis showing downregulation of s*Flt1* levels in MN columns from the electroporated side compared to the non-electroporated one in Hb9-EGFP-misFlt1#2 embryos n = 12, ****P<0.0001. (**n**) Quantification of blood vessel density in MN columns shown as ratio between electroporated and non-electroporated side in Hb9-EGFP-misFlt1#2, n = 8, P=0.0220. Data is represented as mean±s.e.m..

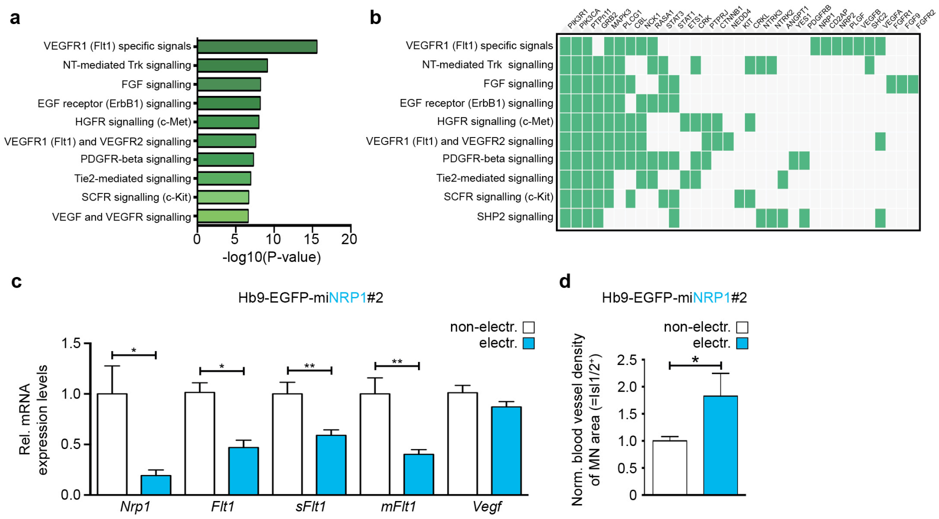

Supplementary Figure 10: Knockdown of NRP1 results in blood vessel ingression into motor neuron columns. (**a**) Enrichment analysis for signalling pathways associated with potential Flt1 regulators that are overexpressed or expressed in E11.5 MNs using *Enrichr*. (**b**) Clustergram of the potential Flt1 regulators grouped by signalling pathways. (**c**) qRT-PCR analysis showing downregulation of *Nrp1*, *Flt1* (*mFlt1* + *sFlt1*), *sFlt1* and *mFlt1* mRNA levels in MN columns of chicken embryos electroporated with a second miRNA for NRP1 (Hb9-EGFP-miNRP1#2). *Vegf* mRNA levels are unaffected. n = 5, *P=0.0212 (*Nrp1*), *P=0.0284 (*Flt1*), **P=0.0035 (*sFlt1*), **P=0.0069 (*mFlt1*), P=ns (*Vegf*).(**d**) Quantification of blood vessel density in MN columns shown as ratio between electroporated and non-electroporated side in Hb9-EGFP-miNRP1#2 electroporated embryos. n = 8, *P=0.0222.

Supplementary Table 1: miRNAs used

Name	Target gene	Target sequence	Location
Hb9-EGFP-miLuc	firefly Luciferase	5'-CGTGGATTACGTCGCCAGTCAA-3'	ORF
Hb9-EGFP-miFlt1#1	mFlt1 and sFlt1	5'-TTACTCTCAGCCATCCATTTG-3'	CDS
Hb9-EGFP-miFlt1#2	mFlt1 and sFlt1	5'-TTCTGCTCTCGCACTGATGGA-3'	CDS
Hb9-EGFP-misFlt1#1	sFlt1	5'-TTGTACAACACAAAGCAATGT-3'	CDS
Hb9-EGFP-misFlt1#2	sFlt1	5'-TTCTGCTCTCGCACTGATGGA-3'	CDS
Hb9-EGFP-miNRP1#1	NRP1	5'-TTGTGTATAATTGCTCGTGTT-3'	CDS
Hb9-EGFP-miNRP1#2	NRP1	5'-TTACTCGGAAGCATGAAGGTA-3'	CDS

Supplementary Table 2: Primers used for plasmid generation: Hb9-sFlt1-HA

Plasmid	Forward	Reverse
mouse Hb9-sFlt1-HA	5'-AGCTAAGCTTATGGTCAGCTGCTGGGACACC-3'	5'ATATGCGGCCGCTTAAGCGTAATCTGGAACATCGTATGGGTAAGCGTAATCTGGAACATCGTATGGGTAAGCGTAATCTGGAACATCGTATGGGTAAAGGATGTCTTCCCCTGTG-3'

Supplementary Table 3: Primers used for qRT-PCR

qRT-PCR primers	Forward	Reverse
mouse *Gapdh*	5'-GGTCCTCAGTGTAGCCCAAG-3'	5'-AATGTGTCCGTCGTGGATCT-3'
mouse *Rpl13a*	5'-GGATCCCTCCACCCTATGACA-3'	5'-CTGGTACTTCCACCCGACCTC-3'
human $Vegf_{165}$	5'-ATCTTCAAGCCATCCTGTGTGC-3'	5'-CAAGGCCCACAGGGCTTTTC-3'
mouse *Vegf*	5'-CGTTCACTGTGAGCCTTGTT-3'	5'-CTTGGCTTGTCACATCTGCA-3'
mouse *Bnip3*	5'-TTTTAAACACCCGAAGCGCA-3'	5'-GAAGTTGTCAGACGCCTTCC-3'
mouse *Pdk1*	5'-TGACCACATTCCGAAGCTCT-3'	5'-AAACCACGCCCAATTAACCC-3'
mouse *Egln3*	5'-CTGACTGTGGTGTGCAATCC-3'	5'-AGAGTGTCTGAAGGTCACCG-3'
mouse N-terminal *Flt1*	5'-CTGACTGTGGTGTGCAATCC-3'	5'-AGAGTGTCTGAAGGTCACCG-3'
mouse *Nrp1*	5'-GCTCTGAAGACCTGGCAATG-3'	5'-GCAGGTTAAAGCTGCAAGGT-3'
chicken *18S*	5'-CGAAAGCATTTGCCAAGAAT-3'	5'-GGCATCGTTTATGGTCGG-3'
chicken *Flt1* *(mFlt1 + sFlt1)*	5'-TGCTGAGGAAGATGCTGGAA-3'	5'-CCATACACGGTGCATGTCAG-3'
chicken *sFlt1*	5'-TCAGAGGTGAGCACTGCAAC-3'	5'-CCTTTAATGTTTTACATTGCTTTGTG-3'
chicken *mFlt1*	5'-CTACAAGCTGCCCCTCACTA-3'	5'-GCGAGGCCAAAATCACAGAT-3'
chicken *Nrp1*	5'-AACTGCGGACTTTTGAACCC-3'	5'-GTTCGCCTGGTCATCATCAC-3'

Supplementary Table 4: Primers used for generating ISH probes

ISH primers	Forward	Reverse
mouse *Sim1*	5'-TATACTGCCTTTGGGGAGAG-3'	5'-CTACCCGTACAACCTTTGTG-3'
mouse *Nse*	5'-CAGTGTGCTGTGATCCCCTT-3'	5'-TAGGGGTGCCTAGTCCTGTC-3'
chicken *Vegfr2*	5'-GATACTCTGAACATTACTTGC-3'	5'-ACACGTGTATCGACCTTT-3'
chicken *Vegf*	5'-TTGTCGAAGGCTGCTCCGG-3'	5'-TCTTGCGATCTCCATCGTGACG-3'
chicken *Nrp1*	5'-TGTCCCTACTGTTTCCGAAGGCAA-3'	5'-TTGCCTGGTTTCCTGGAGATGTCA-3'
